# Higher oxidative balance score was associated with decreased risk of erectile dysfunction: a population-based study

**DOI:** 10.1186/s12937-024-00956-y

**Published:** 2024-05-17

**Authors:** Zhixiao Xu, Weiwei Chu, Xiong Lei, Chengshui Chen

**Affiliations:** 1 Department of Pulmonary and Critical Care Medicine, The First Affiliated Hospital of Wenzhou Medical University, Wenzhou, China; 2 Department of Pulmonary and Critical Care Medicine, The Lu ’an People’s Hospital of Anhui Province, The Lu ’an Hospital Affiliated to Anhui Medical University, Lu ’an, China; 3 Key Laboratory of Interventional Pulmonology of Zhejiang Province, Wenzhou, China; 4 Zhejiang Province Engineering Research Center for Endoscope Instruments and Technology Development, The Quzhou Affiliated Hospital of Wenzhou Medical University, Quzhou People’s Hospital, Quzhou, China

**Keywords:** Oxidative balance score, Erectile dysfunction, Nutrients, Lifestyle

## Abstract

**Background:**

Erectile dysfunction (ED) is a prevalent condition that is thought to be significantly impacted by oxidative stress. The oxidative balance score (OBS) has been built to characterize the state of antioxidant/pro-oxidant balance. There is less known regarding the relationship of OBS with ED.

**Methods:**

This study conducted cross-sectional analyses on 1860 males who participated in the National Health and Nutrition Examination Survey (NHANES) from 2001 to 2004. OBS was constructed by the 16 dietary components and 4 lifestyle factors. Self-reported ED was defined as men who indicated that they “never” or “sometimes” could achieve or keeping an erection adequate for satisfactory intercourse. Multivariate logistic regression models were applied to examine the association between OBS and the risk of ED.

**Results:**

Among 1860 participants, the median OBS was 20 (IQR 15–26), and OBS was lower in males with ED vs. those without ED (*P* = 0.001). The results of our analyses indicated a negative correlation between OBS and ED among male subjects. Specifically, each one-unit increase in the continuous OBS was relate to 3% reduction in the odds of ED after full adjustment. Moreover, when extreme OBS quartiles were compared, the adjusted odds ratio (95% confidence interval) for the 4th OBS category was 0.53 (0.32 to 0.88) after full adjustment (*P* for trend < 0.05). There was also statistical significance in the relationships between dietary/lifestyle OBS with ED, and the association between lifestyle OBS and ED may be even tighter. For each unit increase in lifestyle OBS, the odds of ED decreased by 11% after full adjustment.

**Conclusion:**

Higher OBS was associated with reduced risk of ED in U.S. males. These findings suggested that adopting an antioxidant-rich diet and engaging in antioxidant-promoting lifestyle behaviors may contribute to a lower incidence of ED. These results provided recommendations for a comprehensive dietary and lifestyle antioxidants for ED patients.

**Supplementary Information:**

The online version contains supplementary material available at 10.1186/s12937-024-00956-y.

## Introduction

Erectile dysfunction (ED), predominantly affecting men aged 40 or older, is assumed to one of the most prevalent sexual dysfunctions among adult males [1]. Research based on the National Health and Nutrition Examination Survey (NHANES) reveals that almost one in five males aged 20 or older were affected by ED [2]. The pathogenesis of ED encompasses a range of factors, including psychogenic and organic causes, with many cases involving a combination of both. Vascular ED is the most common among organic disorders, and many studies have indicated a robust association between vascular ED and cardiovascular disease (CVD) [3]. Not only do vascular ED and CVD have similar underlying mechanisms [4, 5] as well as risk factors [6, 7], but they often coexist in the same individual [8].

Oxidative stress is considered a critical factor in CVD [9], with its the pathophysiology involving reactive oxygen species (ROS) [10]. Similarly, the role of ROS in ED has garnered considerable attention [11]. Oxidative stress has been responsible for both neurogenic ED and vascular ED [12]. A complex network of antioxidants and pro-oxidants work together to maintain ROS levels in the body [13, 14], and many antioxidants (e.g., vitamin C and vitamin E) [13] and pro-oxidants (e.g., dietary iron, smoking, and alcohol) [15, 16] are obtained from a range of dietary and non-dietary components. Furthermore, numerous studies over the years have identified that many dietary antioxidants (such as folic acid, calcium, vitamin C, and vitamin E) [17], smoking [18, 19] and alcohol consumption [18, 20, 21] have been implicated in the etiology of ED. Collectively, these findings underscore the importance of oxidative stress in ED and suggest that dietary and lifestyle choices are critical in modulating this risk.

Nonetheless, it is essential to recognize that the effects of anti-/pro-oxidant exposures from dietary and lifestyle on the targeted outcomes may be small and inconsistent [22], but the cumulative impact may be substantial [23]. The oxidative balance score (OBS) is developed by aggregating their respective category scores of antioxidants and prooxidants [24, 25]. And the associations of OBS incorporating multiple dietary and lifestyle exposures with various outcomes were reviewed [26–28], providing insights into the broader implications of oxidative stress. However, few studies have specifically investigated the connection between OBS and ED.

Therefore, to clarify the potential associations between OBS with self-reported ED, we investigated connection between OBS (including dietary and lifestyle OBS) with self-reported ED in a nationally representative sample of males in the U.S.

## Methods

### Participants and study design

Participants were selected from a stratified, multistage cluster probability sampling survey, specifically the NHANES conducted during 2001–2002 and 2003–2004. The NHANES program encompassed a nationally representative sample, with the data available to the public through their website (https://www.cdc.gov/nchs/nhanes/index.htm). To assess the health and nutritional status of human participants, the NHANES studies were conducted every two years. NHANES involving adults and children were approved by the National Center for Health Statistics Ethics Review Board, and all participants provided informed consent.

**Fig. 1:**
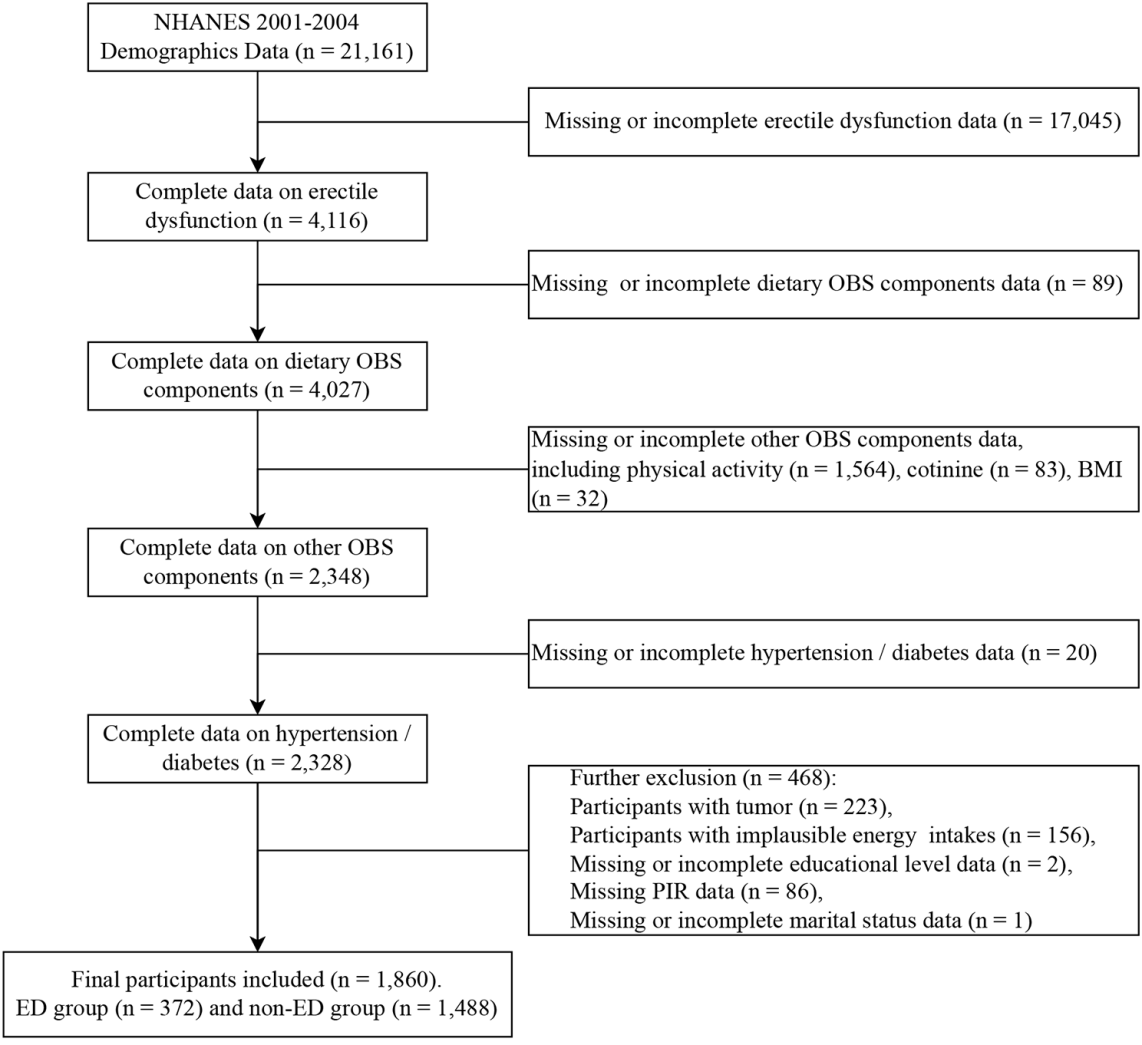
Flowchart design and selection of participants. NHANES, National Health and Nutrition Examination Survey; OBS, oxidative balance score; BMI, body mass index; PIR, poverty income ratio; ED, erectile dysfunction

Exclusion criteria used in the final sample analysis were (1) lack of data on erectile dysfunction, and OBS components (*N* = 18813); (2) lack of data on hypertension or diabetes (*N* = 20); (3) participants with tumor and energy intakes were implausible (more than 4200 kcal/d or less than 800 kcal/d) [25] were excluded (*N* = 379); and (4) lack of data on educational level, poverty income ratio (PIR), and marital status (*N* = 89). From a total of 21161 participants in the NHANES 2001–2004, the final samples included in this analysis were 1860 participants (Fig. 1). Table S1 showed the baseline characteristics of the included and excluded participants.

### Oxidative balance score

We constructed an OBS. The OBS included these antioxidants [including intakes of fiber, carotene, riboflavin, niacin, vitamin B6, total folate, vitamin B12, vitamin C, vitamin E, calcium, magnesium, zinc, copper, and selenium, as well as the weekly metabolic equivalent (MET)] plus prooxidants (the total fat and iron intake, cotinine, BMI, and alcohol intake). These incorporated antioxidant and pro-oxidant exposures were all previously described [25].

Dietary OBS components data were obtained from two 24-h dietary recall interviews collected on two non-consecutive days (the approach, method and content of the two dietary interviews were depicted in detail on the NHANES website). Then, the average food intake during the 2 days could be calculated through the energy, nutrient, and other food components after estimated by the University of Texas Food Intake Analysis System. If participants had only one 24-h dietary recall interview, the average food intake was not calculated. Similarly, to avoid excessive missing values alcohol consumption information was obtained from the 24-h dietary recall interviews.

Cotinine was utilized to reflect smoking, because cotinine has a long half-life and can respond to both active and passive smoking. The value of physical activity was calculated according to the following formula: physical activity (MET- minutes/30d) = MET × frequency in past 30 days × duration of each leisure-time activity over the past thirty days [24].

The scores assigned to each component corresponded to their respective weighted tertile, the rules for the scores assigned to antioxidants and prooxidants were reversed, as shown in Table 1. The total OBS was calculated by summing the scores assigned to each component. Increased OBS represented the predominance of antioxidant exposures, while the opposite was a preference for prooxidants.

**Table 1:** Oxidative balance score (OBS) items and score assignment

OBS items	Property	0	1	2
Dietary OBS components				
Dietary fiber (g/d)	Antioxidant	< 13.00	13.00–20.00	≥ 20.00
Carotene (RE/d)	Antioxidant	< 564.00	564.00-1672.50	≥ 1672.50
Riboflavin (mg/d)	Antioxidant	< 2.02	2.02–2.86	≥ 2.86
Niacin (mg/d)	Antioxidant	< 22.38	22.38–31.34	≥ 31.34
Vitamin B6 (mg/d)	Antioxidant	< 1.66	1.66–2.50	≥ 2.50
Total folate (mcg/d)	Antioxidant	< 346.00	346.00-514.00	≥ 514.00
Vitamin B12 (mcg/d)	Antioxidant	< 3.84	3.84–6.61	≥ 6.61
Vitamin C (mg/d)	Antioxidant	< 41.45	41.45-110.25	≥ 110.25
Vitamin E (ATE) (mg/d)	Antioxidant	< 5.60	5.60–8.64	≥ 8.64
Calcium (mg/d)	Antioxidant	< 680.00	68.00-1101.50	≥ 1101.50
Magnesium (mg/d)	Antioxidant	< 257.00	257.00-358.50	≥ 358.50
Zinc (mg/d)	Antioxidant	< 10.49	10.49–15.66	≥ 15.66
Copper (mg/d)	Antioxidant	< 1.15	1.15–1.59	≥ 1.59
Selenium (mcg/d)	Antioxidant	< 100.90	100.90-144.30	≥ 144.30
Total fat (g/d)	Prooxidant	≥ 110.47	74.47-110.47	< 74.47
Iron (mg/d)	Prooxidant	≥ 20.01	13.74–20.01	< 13.74
Lifestyle OBS components				
Physical activity (MET-minute/30d)	Antioxidant	< 2527.50	2527.50–7440.00	≥ 7440.00
Alcohol (g/d)	Prooxidant	> 11.15	0-11.15	≥ 0
Body mass index (kg/m2)	Prooxidant	≥ 29.28	25.62–29.28	< 25.62
Cotinine (ng/mL)	Prooxidant	≥ 2.73	0.04–2.73	< 0.04

RE, retinol equivalent; ATE, alpha-tocopherol equivalent; MET, metabolic equivalent

### Main outcomes

Self-reported ED was determined using the question inquiring about the ability to achieve and maintain an erection sufficient for satisfactory intercourse: “How would you describe your ability to get and keep an erection adequate for satisfactory intercourse?”. And the Massachusetts Male Aging Study (MMAS) single question was used, as it has been shown to be a validated tool for population-based studies [29]. ED was defined as those who kept the answer options “sometimes able” or “never able”, and non-ED was defined as those who kept the answer options “usually able” or “almost or almost always able” [30–33].

### Assessment of covariates

Potential covariates included age (years), race/ethnicity (including non-Hispanic White, non-Hispanic Black, Mexican American, and other), PIR, education levels (including < high school, high school/general educational development, and > high school), marital status (including with sex partner and without sex partner), hypertension (yes/no), diabetes (yes/no/borderline).

We defined “with sex partner” as those who answered “married” or “living with partner”, and defined “without sex partner” as those who answered “widowed”, “divorced”, “separated” or “never married”.

### Statistical analysis

All analyses followed the principle of complex multi-stage stratified sampling. The individual sample weights were calculated using 1/2 × two-years of interview records (WTINT2YR), as recommended by NHANES. The nonnormal continuous variables were expressed as median [interquartile range (IQR)], while the categorical variables were presented as unweighted frequencies (weighted percentages). And the Wilcoxon test was applied for nonnormal continuous variables, and the Rau-Scott chi-squared test was applied for categorical variables.

Multivariate logistic regression models were performed to investigate the associations between OBS and the diagnosis of ED. In addition to being considered as a continuous variable, the OBS converted to a categorical variable. Univariate logistic regression analysis could initially explore the relationship between OBS and the diagnosis of ED. Moreover, multivariate logistic regression could further eliminate the influence of other confounding factors, thereby determining the correlation between OBS and the diagnosis of ED. To eliminate the effect of demographic factors, Model 2 (adjusted for age, race/ethnicity, PIR, education level, and marital status) was built. To further exclude the effects of hypertension, diabetes, and inflammation, Model 3 (further adjusted for the diagnosis of hypertension and diabetes based on Model 2) and Model 4 (further adjusted for CRP based on Model 3), respectively.

All statistical analyses were conducted using R software (version 4.1.1). To determine statistical significance, a two-sided significance level of *P* < 0.05 was applied.

### Sensitivity analyses

(1) The multivariate logistic regression models were also utilized to evaluate the robustness of the association between the diagnosis of ED and OBS (removing one element at a time from the total score). (2) Exploring the independent relationships of dietary OBS and lifestyle OBS with erectile dysfunction. (3) Subgroup analysis by stratifying factors including marital status, the diagnosis of hypertension and diabetes were performed. (4) The P for interactions were performed.

## Results

### Baseline characteristics

Out of the 1860 men recruited for the study, 372 were ED patients and 1488 were non-ED patients. Table 2 illustrated the differences in the studied variables based on the diagnosis of ED, and significant differences could observe for almost variables. Men with ED had a significantly lower OBS compared to those without ED (*P* = 0.001). According to the results, the age was significantly higher for the ED subjects than for those without ED (*P* < 0.001), whereas the PIR was lower for the ED subjects than for the non-ED subjects (*P* = 0.006). ED subjects were prone to have lower educational level, higher CRP, and more likely have hypertension/diabetes than men without ED. Racial/ethnic differences could not be observed in the prevalence of ED.

**Table 2:** The baseline characteristics of males stratified by the diagnosis of erectile dysfunction: NHANES 2001–2004

Characteristics	All	Erectile dysfunction	Non-erectile dysfunction	*P*
	*N* = 1860	*N* = 372	*N* = 1488	
Age (year)	41 [31;51]	60 [50;70]	40 [30;49]	< 0.001
Race/ethnicity (%)				0.132
Mexican American	350 (6.8%)	82 (7.4%)	268 (6.8%)	
Other Hispanic	59 (4.0%)	14 (5.9%)	45 (3.7%)	
Non-Hispanic White	1039 (75.5%)	222 (77.3%)	817 (75.2%)	
Non-Hispanic Black	340 (9.0%)	47 (6.6%)	293 (9.3%)	
Other Race - Including Multi-Racial	72 (4.8%)	7 (2.8%)	65 (5.1%)	
Poverty income ratio	3.80 [2.18, 5.00]	3.14 [1.76;5.00]	3.90 [2.27;5.00]	0.006
Education (%)				< 0.001
< High school	363 (11.3%)	124 (22.8%)	239 (9.5%)	
High school/general educational development	438 (24.4%)	80 (25.1%)	358 (24.3%)	
> High school	1059 (64.3%)	168 (52.1%)	891 (66.2%)	
Marital status (%)				0.006
ith sex partner	1277 (70.2%)	284 (77.8%)	993 (69.1%)	
Without sex partner	583 (29.8%)	88 (22.2%)	495 (30.9%)	
Hypertension (%)				< 0.001
Yes	484 (22.5%)	189 (46.9%)	295 (18.8%)	
No	1376 (77.5%)	183 (53.1%)	1193 (81.2%)	
Diabetes (%)				< 0.001
Yes	149 (4.9%)	87 (19.5%)	62 (2.7%)	
No	1686 (94.2%)	277 (79.4%)	1409 (96.5%)	
Borderline	25 (0.9%)	8 (1.1%)	17 (0.8%)	
C-reactive protein (mg/dL)	0.14 [0.06, 0.30]	0.21 [0.11, 0.40]	0.13 [0.06, 0.29]	< 0.001
Oxidative balance score	20 [15, 26]	18 [13, 24]	21 [15, 26]	0.001
Category				0.003
Q1 (< 15)	500 (24.4%)	122 (32.2%)	378 (23.3%)	
Q2 (15 to 20)	437 (23.7%)	96 (26.6%)	341 (23.3%)	
Q3 (20 to 26)	463 (24.8%)	91 (23.5%)	372 (25.0%)	
Q4 (≥ 26)	460 (27.0%)	63 (17.7%)	397 (28.5%)	

### The association between erectile dysfunction and oxidative balance score

A negative correlation between OBS and ED could be observed (Table 3), and the association was stable in the different models. An increase of one unit in OBS was related to a 4% reduction in the odds of ED in the unadjusted model, and a 3% reduction in Model 4. And one standard deviation increase in OBS was linked to a reduced ED risk [adjusted odds ratio (OR) (95% confidence interval (CI)): 0.82 (0.69 to 0.99)].

Moreover, when compared to the 1st quartile of OBS, participants with the 4th OBS exhibited a significantly lower risk of ED (adjusted OR 0.53; 95% CI 0.32–0.88). This finding was consistent across all models, although only the 4th quartile OBS remained statistically significant. Furthermore, the observed trend was significant in all models, with *P*-values less than 0.05.

**Table 3:** Odds ratios (95% CI) for the association between the oxidative balance score and erectile dysfunction

	Model 1			Model 2			Model 3			Model 4	
	OR (95%CI)	*P* value		OR (95%CI)	*P* value		OR (95%CI)	*P* value		OR (95%CI)	*P* value
OBS	0.96 (0.94 to 0.98)	0.001		0.97 (0.95 to 0.99)	0.012		0.97 (0.95 to 0.997)	0.031		0.97 (0.95 to 0.998)	0.039
OBS per SD	0.76 (0.65 to 0.89)	0.001		0.80 (0.67 to 0.95)	0.012		0.82 (0.68 to 0.98)	0.031		0.82 (0.69 to 0.99)	0.039
Q1	Reference	Reference		Reference	Reference		Reference	Reference		Reference	Reference
Q2	0.83 (0.61 to 1.12)	0.202		0.79 (0.57 to 1.11)	0.161		0.82 (0.59 to 1.14)	0.225		0.82 (0.59 to 1.15)	0.236
Q3	0.68 (0.43 to 1.08)	0.098		0.72 (0.39 to 1.34)	0.283		0.76 (0.41 to 1.39)	0.343		0.76 (0.41 to 1.40)	0.352
Q4	0.45 (0.29 to 0.71)	0.001		0.50 (0.31 to 0.80)	0.006		0.52 (0.32 to 0.86)	0.013		0.53 (0.32 to 0.88)	0.017
*P* for trend		0.001			0.013			0.025			0.029

OR, odds ratio; CI, confidence intervals; OBS, oxidative balance score. Model 1 was a crude model. Model 2 adjusted for age, race/ethnicity, education, poverty income ratio, and marital status. Model 3 further adjusted for the diagnosis of hypertension and diabetes based on Model 2. Model 4 additionally adjusted for CRP based on Model 3

### Exploring the independent relationships of dietary OBS and lifestyle OBS with erectile dysfunction

The potential impact of both dietary OBS and lifestyle OBS on ED cannot be neglected. With regard to dietary OBS, there was a notable correlation between OBS and the risk of ED in Model 1 [OR (95% CI): 0.96 (0.94–0.98)] and in Model 2 [adjusted OR (95% CI): 0.97 (0.95–0.998)]. But the association did not maintain statistically significant in Model 3 and Model 4. For lifestyle OBS, an increased OBS was related to a lower risk of ED [adjusted OR (95% CI): 0.89 (0.79–0.99)] after full adjustment, even if the association was subtle in Model 1 (Table 4).

**Table 4:** Odds ratios (95% CI) for the association between the dietary OBS/lifestyle OBS and erectile dysfunction

	Dietary OBS			Lifestyle OBS		*P* for interaction
	OR (95%CI)	*P* value		OR (95%CI)	*P* value	
Model 1	0.96 (0.94 to 0.98)	0.001		0.95 (0.88 to 1.02)	0.171	0.441
Model 2	0.97 (0.95 to 0.998)	0.037		0.87 (0.78 to 0.96)	0.009	0.684
Model 3	0.98 (0.95 to 1.002)	0.065		0.88 (0.79 to 0.99)	0.033	0.603
Model 4	0.98 (0.95 to 1.003)	0.079		0.89 (0.79 to 0.99)	0.038	0.615

OR, odds ratio; CI, confidence intervals; OBS, oxidative balance score. Model 1 was a crude model. Model 2 further adjusted for age, race/ethnicity, education, poverty income ratio, and marital status. Model 3 further adjusted for the diagnosis of hypertension and diabetes based on Model 2. Model 4 additionally adjusted for CRP based on Model 3

### Sensitivity analyses

In sensitivity analyses, after removing from the score one at a time with the original OBS, the odds ratios did not significantly affect after full adjustment (Table 5). This suggests that the overall robustness of the model was maintained. Since BMI and physical activity seemed to have an impact on the association between OBS and ED, but BMI and physical activity were already incorporated into OBS and lifestyle OBS, further subgroup analyses stratified by BMI and physical activity were performed only in the association between dietary OBS and ED (Table S2). After adjustment, there was a notable correlation between OBS and the risk of ED among participants with among participants with BMI < 25.62 kg/m^2^ [adjusted OR (95% CI): 0.94 (0.89–0.99)], and *P* for interaction < 0.05. But this association was not statistically significant between OBS and ED stratified by physical activity.

**Table 5:** Sensitivity analyses to evaluate the impact of individual oxidative balance score components on the oxidative balance score

	OR (95%CI)	*P* value
OBS excluding dietary fiber	0.97 (0.94 to 0.997)	0.031
OBS excluding carotene	0.97 (0.95 to 0.996)	0.027
OBS excluding riboflavin	0.97 (0.94 to 0.999)	0.045
OBS excluding niacin	0.97 (0.95 to 0.9997)	0.048
OBS excluding vitamin B6	0.97 (0.94 to 0.999)	0.042
OBS excluding total folate	0.97 (0.94 to 0.999)	0.043
OBS excluding vitamin B12	0.97 (0.94 to 0.997)	0.034
OBS excluding vitamin C	0.97 (0.95 to 0.998)	0.034
OBS excluding vitamin E	0.97 (0.94 to 0.997)	0.031
OBS excluding calcium	0.97 (0.95 to 0.9997)	0.048
OBS excluding magnesium	0.97 (0.94 to 0.999)	0.041
OBS excluding zinc	0.97 (0.94 to 0.998)	0.040
OBS excluding copper	0.97 (0.94 to 0.999)	0.045
OBS excluding selenium	0.97 (0.94 to 0.999)	0.041
OBS excluding total fat	0.98 (0.95 to 0.998)	0.036
OBS excluding iron	0.98 (0.95 to 0.998)	0.037
OBS excluding physical activity	0.98 (0.95 to 1.003)	0.083
OBS excluding alcohol	0.97 (0.95 to 0.996)	0.027
OBS excluding body mass index	0.97 (0.95 to 1.00)	0.051
OBS excluding cotinine	0.97 (0.95 to 0.998)	0.037

OR, odds ratio; CI, confidence intervals; OBS, oxidative balance score. Model adjusted for age, race/ethnicity, education, poverty income ratio, marital status, the diagnosis of hypertension and diabetes and CRP

There were 335 ED subjects aged 40 and older, but there were only 37 ED subjects with ages ranging from 20 to 40 years. We did not stratify by age because the numbers were so small. After full adjustment, the OBS was negatively associated with ED participants among those with sex partners (OR, 0.97; 95% CI, 0.94–0.99), and those without diabetes (OR, 0.97; 95% CI, 0.94–0.997). No significant interaction was detected (*P* for interaction > 0.05) (Table S3). Moreover, the OBS was also negatively associated with ED participants among those without hypertension in Model 1 and Model 2, but this association was not statistically significant after further adjustment.

## Discussion

In the present study, we examined the relationship of OBS with ED and observed that increased OBS was associated with a reduced risk of ED. This negative association persisted in dietary OBS and lifestyle OBS, although the latter appeared to have a more significant impact after full adjustment. These findings highlight the potential role of OBS in reducing the risk of ED, indicating that maintaining a balanced oxidative environment through dietary and lifestyle changes may be a beneficial strategy for preventing and managing ED.

The composition of the OBS encompasses 20 components, many of which have demonstrated significant involvement ED. Calcium and vitamin E influence pathways related to nitric oxide (NO) release, which is integral to the pathophysiology of ED [17]. Vitamin C had a direct impact on the biological activity of endothelial nitric oxide synthase (NOS) [34]. Smoking, both active and passive, could increase the risk of ED [19] by mechanisms that mainly included endothelial impairment, reduction in the availability of NO, and oxidative stress [35]. Alcohol intake was associated with reduced ED in certain populations [18, 20], potentially attributable to the antioxidant properties of red wine, although excessive alcohol intake has been correlated with reduced NO release in mouse cavernosum [36]. Nevertheless, alcohol abuse is cautioned against due to numerous studies indicating a heightened prevalence of sexual dysfunction among alcohol-dependent individuals compared to the general populace [21]. Obesity was an independent risk factor for ED [37], while bariatric surgery could elevate NOS expressions and intracavernosal pressure, particularly evidenced in Otsuka Long-Evans Tokushima fatty rats [38]. Daily moderate exercise, recognized as an important stimulant of vascular NO production [39], most strongly correlated with ED [17].

In our study, lifestyle OBS seemed to be more important compared to dietary OBS. A one-unit increase in lifestyle OBS was associated with an 11% reduction in the incidence of ED after full adjustment. Moreover, in sensitivity analyses, BMI seemed to have an impact on the relationship between dietary OBS and ED. Derby et al. demonstrated that physical activity could mitigate the risk of ED even in early middle age, indicating that adopting a healthy lifestyle early on could be a key strategy for mitigating the burden of ED [40].

A range of factors act and interact in influencing the risk of ED, and among these factors, oxidative stress, diet, and lifestyle could not be ignored. In comparison to isolated individual factors, a composite metric encompassing both pro-oxidants and antioxidants is posited to exhibit a more robust correlation with health-related outcomes [26]. Therefore, OBS was adopted as a comprehensive gauge of oxidative stress-related exposure. Our findings indicated that mitigating the risk of ED necessitated a comprehensive consideration of both oxidative and antioxidant profiles across multiple constituents, rather than relying solely on individual components.

In our investigation, no statistically significant interaction by diabetes status was discernible. It was widely documented that diabetic males were more prone to suffer from ED than non-diabetic males [31]. However, in non-diabetic patients, the correlation between OBS and ED was stronger than in diabetics patients. This suggested that diabetes might complicate the relationship between oxidative balance and ED. Diabetic men afflicted with ED presented a challenging clinical population due to their often-refractory response to traditional medication [41]. In this complex population, a more comprehensive and precise measure of redox status might be needed.

The study had several limitations. First, Although the NHANES data collected through a complex multi-stage probability sampling design were representative of the sample of the ambulatory civilian population, limitations of data remained. We did not include medication intake in the analysis because of the high number of missing values for medication intake. Moreover, limited by the cross-sectional nature of the data, the relationship of OBS with ED was difficult to infer causation and only correlations could be concluded. Second, the OSB approach needed further refinement. Ours was not the first time to attempted to construct an OBS using this approach [24, 25], but there was disagreement about the appropriateness of using tertiles to construct scores, particularly for exposures that induce threshold effects [42]. The dietary pro-oxidants/anti-oxidants included in this study were not comprehensive, future studies should warrant further investigation and possible inclusion of other components of OBS, and endogenous components of the oxidative stress pathway should also deserve attention [15]. Third, there were no OS biomarkers available to validate the validity of OBS.

## Conclusion

Our findings revealed a robust negative association between elevated OBS and the risk of ED, implying that the predominance of antioxidant exposures is likely to be protective against the development of ED. Importantly, our results underscore the significance of adopting an integrated approach towards oxidative homeostasis, transcending the singular focus on individual antioxidant components. Furthermore, a lifestyle focused on antioxidants may be the best way to reduce the burden of ED on the health and well-being of older men.

### Electronic supplementary material

Below is the link to the electronic supplementary material.

Supplementary Material 1

## Data Availability

Data could be publicly available from the website (https://www.cdc.gov/nchs/nhanes/index.htm).
