# Supplementary material for: Higher oxidative balance score was associated with decreased risk of erectile dysfunction: a population-based study
Source: Nutr J. 2024 May 17;23:54. doi: 10.1186/s12937-024-00956-y (PMC11102141; doi:10.1186/s12937-024-00956-y)
Supplement: Supplementary file 1 — Supplementary Material 1 [file 12937_2024_956_MOESM1_ESM.docx]

Table S1. The baseline characteristics of included and excluded participants.

| Characteristics | All | Excluded participants | Included participants | P overall |
| --- | --- | --- | --- | --- |
|  | N=21161 | N=19301 | N=1860 |  |
| Gender (%) |  |  |  | <0.001 |
| Male | 10301 (48.7%) | 8441 (39.5%) | 1860 (100%) |  |
| Female | 10860 (51.3%) | 10860 (60.5%) | 0 (0.00%) |  |
| Age (year) | 35 [17;51] | 32 [14;51] | 41 [31;51] | <0.001 |
| Race/ethnicity (%) |  |  |  | <0.001 |
| Mexican American | 5295 (8.9%) | 4945 (9.3%) | 350 (6.8%) |  |
| Other Hispanic | 858 (5.3%) | 799 (5.5%) | 59 (4.0%) |  |
| Non-Hispanic White | 8739 (68.3%) | 7700 (67.0%) | 1039 (75.5%) |  |
| Non-Hispanic Black | 5344 (12.1%) | 5004 (12.6%) | 340 (9.0%) |  |
| Other Race - Including Multi-Racial | 925 (5.5%) | 853 (5.6%) | 72 (4.8%) |  |

Table S2. The association between dietary oxidative balance score and erectile dysfunction stratified by body mass index and physical activity.

|  | Model 1 | | |  | Model 2 | | |
| --- | --- | --- | --- | --- | --- | --- | --- |
|  | OR (95%CI) | P value | P for interaction |  | OR (95%CI) | P value | P for interaction |
| Body mass index (kg/m2) |  |  | 0.006 |  |  |  | 0.024 |
| ≥29.28 | 0.96 (0.94 to 0.98) | 0.001 |  |  | 0.97 (0.94 to 1.00) | 0.055 |  |
| 25.62-29.28 | 1.00 (0.96 to 1.04) | 0.913 |  |  | 1.04 (0.98 to 1.10) | 0.181 |  |
| <25.62 | 0.93 (0.90 to 0.97) | <0.001 |  |  | 0.94 (0.89 to 0.99) | 0.026 |  |
| Physical activity (MET-minute/30d) |  |  | 0.373 |  |  |  | 0.377 |
| <2527.50 | 0.98 (0.94 to 1.02) | 0.380 |  |  | 1.00 (0.94 to 1.05) | 0.889 |  |
| 2527.50-7440.00 | 0.95 (0.92 to 0.99) | 0.007 |  |  | 0.97 (0.93 to 1.02) | 0.210 |  |
| ≥7440.00 | 0.95 (0.91 to 0.99) | 0.009 |  |  | 0.96 (0.91 to 1.01) | 0.107 |  |

OR, odds ratio; CI, confidence intervals.

Model 1 was a crude model. Model 2 further adjusted for age, race/ethnicity, education, poverty income ratio, marital status, the diagnosis of hypertension and diabetes, and CRP.

The basis for stratification of BMI by and physical activity was according to the values in Table 1.

Table S3. Subgroup analysis of the association between oxidative balance score and erectile dysfunction.

|  | Model 1 | |  | Model 2 | |  | Model 3 | |  | Model 4 | |
| --- | --- | --- | --- | --- | --- | --- | --- | --- | --- | --- | --- |
|  | OR (95%CI) | P value |  | OR (95%CI) | P value |  | OR (95%CI) | P value |  | OR (95%CI) | P value |
| **Marital status** |  |  |  |  |  |  |  |  |  |  |  |
| With sex partner | 0.95 (0.93 to 0.98) | <0.001 |  | 0.96 (0.94 to 0.99) | 0.004 |  | 0.96 (0.94 to 0.99) | 0.009 |  | 0.97 (0.94 to 0.99) | 0.018 |
| Without sex partner | 0.98 (0.93 to 1.03) | 0.398 |  | 0.996 (0.94 to 1.05) | 0.879 |  | 1.001 (0.95 to 1.06) | 0.981 |  | 0.997 (0.94 to 1.05) | 0.908 |
| P for interaction | | 0.307 |  |  | 0.221 |  |  | 0.207 |  |  | 0.207 |
| **Diabetes** |  |  |  |  |  |  |  |  |  |  |  |
| Yes | 0.99 (0.93 to 1.05) | 0.771 |  | 1.002 (0.93 to 1.08) | 0.968 |  | 0.99 (0.91 to 1.07) | 0.800 |  | 0.998 (0.92 to 1.08) | 0.957 |
| No | 0.96 (0.94 to 0.99) | 0.007 |  | 0.96 (0.93 to 0.99) | 0.018 |  | 0.97 (0.94 to 0.996) | 0.027 |  | 0.97 (0.94 to 0.997) | 0.032 |
| P for interaction | | 0.289 |  |  | 0.159 |  |  | 0.278 |  |  | 0.276 |
| **Hypertension** | |  |  |  |  |  |  |  |  |  |  |
| Yes | 0.97 (0.94 to 0.999) | 0.044 |  | 0.98 (0.94 to 1.03) | 0.389 |  | 0.97 (0.93 to 1.02) | 0.277 |  | 0.98 (0.93 to 1.03) | 0.433 |
| No | 0.97 (0.94 to 0.99) | 0.019 |  | 0.97 (0.93 to 1.004) | 0.080 |  | 0.97 (0.93 to 1.01) | 0.139 |  | 0.97 (0.93 to 1.01) | 0.152 |
| P for interaction | | 0.864 |  |  | 0.653 |  |  | 0.882 |  |  | 0.880 |

OR, odds ratio; CI, confidence intervals.

Model 1 was a crude model. Model 2 further adjusted for age, race/ethnicity, education, poverty income ratio, and marital status, except the stratification factor itself. Model 3 further adjusted for the diagnosis of hypertension and diabetes, except the stratification factor itself. Model 4 additionally adjusted for CRP.
